# Effect of positive end-expiratory pressure on ultrasound measurement of optical sheath nerve diameter: preliminary study

**DOI:** 10.1186/cc12263

**Published:** 2013-03-19

**Authors:** T Aslanidis, E Anastasiou, E Geka, E Efthimiou, A Myrou, A Kontos, D Papadopoulos, E Boultoukas, M Giannakou-Peftoulidou

**Affiliations:** 1 A.H.E.P.A. University Hospital, Thessaloniki, Greece

## Introduction

Non-invasive ocular ultrasonography has recently been proposed to detect elevated intracranial pressure (ICP). On the other hand, the effect of positive end-expiratory pressure (PEEP) on ICP is well documented. The aim of the present ongoing study is to record the effect of changes of airway pressures on optical sheath nerve diameter (OSND).

## Methods

In this prospective observational study, measurements of OSND were carried out in 21 patients of a polyvalent 10-bed adult ICU before and 2 minutes after changes of PEEP from a baseline of 5 with 1 cmH_2_O increments, to a maximum of 12. Two measurements were performed with a 7.5 MHz probe (MicroMaxx; Fujifilm Sonosite Inc., USA) in each eye before and after PEEP changes. Demographic data (APACHE II score, age, sex, diagnosis) Ppeak and Pm were also recorded. Exclusion criteria were intracranial pathology, ocular neuritis and trauma. Normality with P-P plots and the Kolmogorov-Smirnov test were calculated followed by bivariate and single linear regression analysis (IBM SPPS v17, significance *P <*0.05).

## Results

Seventy-two measurements were included for further analysis. Mean ± SD values were: PEEP (7.9 ± 2.3), Ppeak (24.35 ± 5), Pm (12.03 ± 3.16), OSNDright (4.03 ± 0.79), OSNDleft (4.1 ± 0.79), ΔOSND (0.24 ± 0.2), while values for age and APACHE II score were 62 ± 4 years and 14.4 ± 1.4 respectively. PEEP and OSND seem to have a moderate relation (tendency equation: OSND = 2.454 + 0.581×PEEP) compared with the weaker effect of Ppeak and Pm (Table 1).

## Conclusion

Our study identified a moderate relation between PEEP and OSND and a weaker one between Ppeak, Pm and OSND. Thus, in selected cases OSND could serve as a bedside marker of effect of airway pressure to ICP. Yet, larger studies are needed to come to a safer conclusion.

**Table 1:** Correlations (*r*^2^) between airway pressures and OSND

	OSNDright	OSNDleft
PEEP	0.581*	0.571*
Ppeak	0.467*	0.448*
Pm	0.491**	0.467**

*P <0.01, **P <0.05.
